# Effect of a Gelatin-Based Film Including *Gelidium* sp. Algal Flour on Antimicrobial Properties Against Spoilage Bacteria and Quality Enhancement of Refrigerated *Trachurus trachurus*

**DOI:** 10.3390/foods14091465

**Published:** 2025-04-23

**Authors:** Antonio Gómez, Lucía López, José M. Miranda, Marcos Trigo, Jorge Barros-Velázquez, Santiago P. Aubourg

**Affiliations:** 1 Department of Food Technology, Marine Research Institute (CSIC), 36208 Vigo, Spain; tonygn74@gmail.com (A.G.); lucialpzlpz@hotmail.com (L.L.); mtrigo@iim.csic.es (M.T.); 2 Department of Analytical Chemistry, Nutrition and Food Science, School of Veterinary Sciences, University of Santiago de Compostela, 27002 Lugo, Spain; jorge.barros@usc.es

**Keywords:** *Gelidium* sp., flour, gelatin film, food packaging, horse mackerel, refrigeration, microbial activity, trimethylamine, lipid damage

## Abstract

Background: Red macroalgal flour resulting from commercial phycocolloid extraction has been shown to include valuable preservative compounds. Methods: This study focused on the preservative effect of a gelatin-based packaging system including *Gelidium* sp. flour during refrigerated storage of *Trachurus trachurus* fillets. Different microbial and chemical indices related to quality loss were determined in fish muscle during a 6-day storage period at 4 °C. Results: Compared with gelatin-packaged control samples, those packaged in the system including the algal flour presented significantly lower microbial development (aerobic bacteria, psychrotrophic bacteria, and proteolytic bacteria) and significantly lower chemical indices related to microbial development (pH and trimethylamine). With respect to lipid oxidation, there was significantly greater retention of peroxides and significantly lower formation of secondary oxidation products in the samples packaged with the algal flour. Additionally, the algal flour group presented significantly less lipid hydrolysis. Conclusions: A preservative effect was derived from the addition of *Gelidium* flour to a gelatin-based packaging system during refrigerated storage of *T. trachurus*. This study supports the practical and valuable use of *Gelidium* sp. flour and addresses the current global interest in natural sources of preservative compounds and the use of marine byproducts.

## 1. Introduction

Seafood rapidly deteriorates postmortem because of a variety of biochemical and microbial breakdown pathways [1,2,3]. The most common way to delay spoilage and loss of freshness in seafood is by reducing storage temperatures through the addition of ice or mechanical refrigeration. However, neither method is fully effective in ensuring that seafood quality is maintained over long periods of time or if there are breaks in the cold chain. Different strategies have been developed to increase the shelf-life of refrigerated marine species [4,5,6,7]. Recently, the use of packaging films containing preservative compounds has received much attention [8,9]. Among preservative compounds, antioxidants and antimicrobials derived from natural sources have been recommended as safer alternatives to synthetic ones, given that adverse health effects can result from the persistent consumption of synthetic compounds [10,11,12]. Therefore, the identification and isolation of novel natural preservatives has attracted considerable attention in recent decades.

Processing marine species results in the generation of large amounts of byproducts. Among the currently most utilized marine byproducts, fish meal, oil, silage, and fertilizer production can be mentioned [13,14]. The valorization of byproducts from the seafood processing industry has been a hot topic in recent years because such byproducts contain the same valuable components as the commercial parts of marine species [15,16]. Indeed, numerous bioactive compounds present in marine byproducts can be incorporated into nutraceuticals, functional food formulations, and/or pharmaceuticals [3,17,18]. The development of sustainable technologies for the recovery, purification, and identification of high value-added compounds can promote better utilization of byproducts and lead to profitable use in food packaging and other sustainable food strategies [19,20,21,22].

Seaweed has been used as part of the ancient human diet in a wide range of Asian countries. More recently, seaweed intake has gained consumer interest in Western countries because of the numerous benefits to human health derived from its regular intake [23]. Marine macroalgae contain chemical constituents with significant antimicrobial and antioxidant properties [23,24,25]. Among macroalgae, red macroalgae have constituents that support their high nutritional value [26,27]. Specifically, previous works have shown that red algae contain significant amounts of compounds that have potential preservative activity (phenolic compounds, flavonoids, carbohydrates, and others), which have the capacity to improve the quality of fishery products because of their antioxidant activities [28,29] and potential antimicrobial activities [30,31]. Traditionally, within the food industry, red algae have been used as sources of phycocolloids (algin, furcellaran, agar, and carrageenan) [30,32]. These compounds have been used by the food industry as thickeners and stabilizers, and as components of edible films in new food packaging applications [33,34]. Algae-based films are biodegradable and have distinct visual characteristics, such as transparency, which are essential for applications in food packaging and coating. Packaging materials for seaweed-based foods must comply with general safety regulations to protect the health of consumers, such as the European Union regulation EU 10/2011 regarding food contact materials [35]. The market size of the global seaweed packaging industry was valued at USD 699.23 million in 2023 and is expected to expand at a compound annual growth rate of 6.6% from 2024 through 2030 [36].

This current work focuses on the use of flour derived from the red alga *Gelidium* sp. as a source of preservative compounds for the quality retention of refrigerated fish. In previous studies, aqueous *Gelidium* sp. flour extracts were shown to have significant antimicrobial [37,38] and antioxidant [37,39] effects on different kinds of seafood. In the present work, the effect of the incorporation of *Gelidium* sp. flour into a gelatin film was studied during a 6-day refrigerated storage period of Atlantic horse mackerel (*Trachurus trachurus*) fillets. Throughout this period, different microbial and chemical indices related to quality loss were assessed.

## 2. Materials and Methods

### 2.1. Initial Algal Flour and Biopolymer Film Preparation

All solvents and chemical reagents used throughout this work were reagent grade (Merck, Darmstadt, Germany), unless otherwise indicated. *Gelidium* sp. flour was obtained from Industrias Roko S. A. (Llanera, Asturias, Spain), with a proximate composition determined following AOAC procedures [40].

Teleost gelatin films (Sigma, Life Sciences, Steinheim, Germany) were obtained by casting from their film-forming solutions (FFSs) following the procedure described by Stejskal et al. [41]. Oxidized sodium alginate (OSA) was prepared as described by Balakrishnan et al. [42].

For the preparation of the biopolymer film made from the combination of gelatin and algal flour, 50 g of a dry mixture of algal flour–gelatin (5:95, *w*/*w*) was dissolved in 500 mL of 0.01 M NaOH. The obtained mixture was stirred for 20 min at 40 °C. Subsequently, OSA (2.5 g; 5 wt.%) and glycerol (7.5 g; 30 wt.%) were added to the mixture as a cross-linking agent and plasticizer, respectively. The resulting suspension was stirred at 40 °C for 120 min. The FFSs were then cast on Teflon-coated trays and dried in a convection oven at 40 °C for 48 h. The obtained films, referred to as red algal flour (RAF) packaging films, were subsequently conditioned in a chamber at 4 ± 1 °C for 48 h before use. Simultaneously, a control gelatin film without algal flour was prepared in the same manner as the RAF packaging film, which was referred to as the control packaging film (CTR). The algal meal–gelatin ratio chosen for the present study was established on the basis of previous trials conducted in our laboratory. The seaweed meal–gelatin ratio of 5:95 represented the highest possible concentration of seaweed meal that did not significantly affect the sensory and physical characteristics of the fish muscle portions.

### 2.2. Raw Fish, Packaging, and Sampling

Fresh *T. trachurus* samples (*n* = 45) were caught in June 2024 by a local fishery company near the Galician Atlantic coast (northwestern Spain) and transported to the laboratory on ice in less than 10 h. The length and weight of the fish samples were 35–38 cm and 385–445 g, respectively. Upon arrival at the laboratory, nine individual fish samples were analyzed on day 0. These nine fish were divided into three different groups (three fish per group). All white muscle samples from the dorsal location were analyzed in triplicate. Another 36 remaining fish samples were distributed into two groups of 18 different specimens per group, which were filleted and cut into pieces of approximately 35 g each, packed in CTR or RAF systems, and stored in a refrigerated chamber (4 °C) for 6 days.

Sampling and analysis were carried out on days 2 and 6 of storage. At each sampling time, 18 samples were taken from each group for analysis, and the white muscle was examined independently.

Portions of 10 g of fish muscle were aseptically dissected and mixed with 90 mL of 0.1% peptone water and then homogenized in sterilized stomach bags (AES, Combourg, France) for 2 min at maximum power. From this homogenate, serial dilutions of the microbial extracts were prepared in 0.1% peptone water.

The total number of aerobic microorganisms was determined via plate count agar (PCA) (Oxoid Ltd., London, UK). After incubation at 30 °C for 48 h, all the colonies obtained were considered total aerobic microorganisms. Total psychotropic microorganisms were determined via PCA and incubated at 7–8 °C for 7 days, after which all the colonies obtained were considered total psychotropic microorganisms. Enterobacteriaceae in the coliform group were investigated on violet red bile agar (VRBA). After incubation at 37 ± 0.5 °C for 24 h, all pink to red colonies obtained were considered to belong to this group. Microorganisms exhibiting proteolytic or lipolytic phenotypes were investigated on casein agar or tributyrin agar, respectively, following incubation at 30 °C for 48 h. All colonies presenting transparent halos were considered proteolytic (casein agar) or lipolytic (tributyrin agar), respectively. In all cases, analyses were performed in triplicate, and bacterial counts were transformed into log colony-forming units (CFUs) g^−1^ muscle before being subjected to statistical analysis.

### 2.3. Determination of Chemical Indices Related to Quality Loss

The evolution of pH in the *T. trachurus* muscle during storage time was determined by means of a 6 mm diameter insertion electrode (Crison, Barcelona, Spain).

The trimethylamine (TMA) content was determined via the picrate spectrophotometric (410 nm) method (Beckman Coulter DU640 spectrophotometer, Brea, CA, USA), as described previously by Tozawa et al. [43], and the results are presented as mg TMA-N·kg^−1^ muscle.

Lipids from *T. trachurus* white muscle were extracted following the Bligh and Dyer [44] method. This method involves single-phase solubilization of lipids via a chloroform–methanol (1:1) mixture. The results are presented as g lipid·kg^−1^ muscle.

The free fatty acid (FFA) content was determined from the lipid extract of the fish muscle via the Lowry and Tinsley [45] method, which involves the formation of a complex with cupric acetate–pyridine followed by spectrophotometric (715 nm) assessment. The results are expressed as g FFAs·kg^−1^ lipids.

The peroxide content of the lipid extract of *T. trachurus* muscle was determined spectrophotometrically (520 nm) on the basis of the methods reported by Chapman and McKay [46]. In brief, peroxides included in the lipid extract are reduced with ferric thiocyanate. The results are expressed as mEq active oxygen·kg^−1^ lipids.

The thiobarbituric acid index (TBA-i) was determined according to the method developed by Vyncke [47]. This method involves the reaction of a trichloroacetic acid extract of fish muscle with thiobarbituric acid. Thus, the thiobarbituric acid reactive substance (TBARS) content was determined spectrophotometrically at a wavelength of 532 nm. To obtain a quantitative evaluation, a standard curve of 1,1,3,3-tetraethoxy-propane was previously constructed. The results are presented as mg malondialdehyde·kg^−1^ muscle.

The content of fluorescent compounds was measured in the lipid extract of *T. trachurus* muscle at 393/463 and 327/415 nm using an LS 45 Fluorimeter (Perkin Elmer España, Tres Cantos, Madrid, Spain) [48].

### 2.4. Statistical Analysis

The data (average value ± standard deviation) obtained from all microbiological and chemical analyses were subjected to analysis of variance (ANOVA) to explore differences resulting from the effects of the packaging conditions and the storage time. The average values obtained were compared via the least-squares difference (LSD) method. For all the cases, analyses were carried out via PASW Statistics 18 software for Windows (Statistica version 6.0, 2002; Statsoft Inc., Tulsa, OK, USA). A *p*-value < 0.05 was considered to indicate a statistically significant difference.

## 3. Results

### 3.1. Bacterial Evolution in Refrigerated Fish

**Figure 1:**
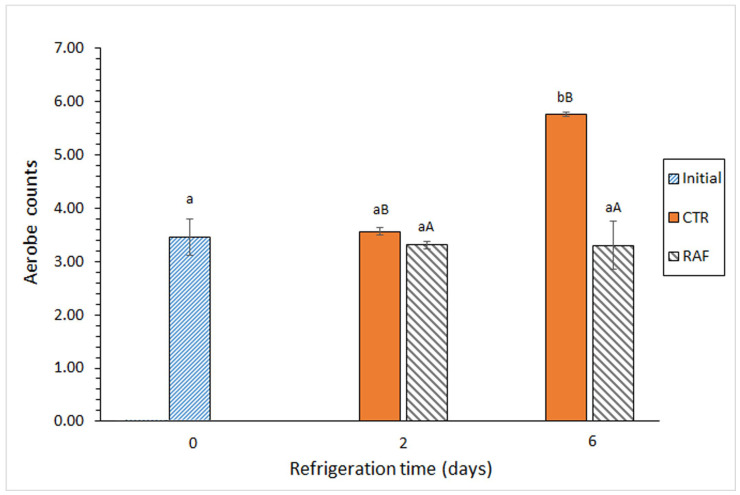
Evolution of aerobic bacteria counts (log CFU·g^−1^ muscle) in refrigerated *Trachurus trachurus* subjected to different packaging conditions. Average values of three replicates (*n* = 3). Standard deviations are indicated by bars. For each packaging condition, different lowercase letters (a, b) indicate significant differences with respect to the refrigeration time; at each refrigeration time, different capital letters (A, B) indicate significant differences with respect to the packaging condition. Packaging conditions: CTR (control; gelatin-packaging condition) and RAF (red alga flour; combined gelatin packaging and alga flour extract treatment), as described in the Section 2.

In the control group, the aerobic bacteria (Figure 1) and *Enterobacteriaceae* (Table 1) counts increased significantly as the storage time increased. In contrast, in the treatment group, these counts did not change significantly over time. Notably, a comparative analysis of the aerobic bacteria between the groups revealed a significant difference at the end of the experiment; it reached 2.46 log CFU·g^−1^ muscle (Figure 1).

In the case of psychrotrophic bacteria and specific spoilage organisms, such as proteolytic and lipolytic bacteria, there were significant increases in both the treatment and control groups as the storage time increased (Table 1). Comparisons between the treatment and control groups did not reveal significant differences for any of these bacterial groups at the end of the experiment; however, on day 2, the average psychrotrophic and proteolytic bacteria counts were lower in the treatment group than in the control group. The difference reached 1.46 and 0.49 log CFU·g^−1^ muscle for psychrotrophic and proteolytic bacteria, respectively (Table 1). Finally, the number of lipolytic bacteria was greater in the control group than in the treated group, but the differences were not significant.

### 3.2. Evolution of Chemical Parameters Related to Microbial Activity in Refrigerated Fish

The pH increased significantly with increasing storage time in both groups (Table 2). However, at the end of the experiment, the pH of the treatment group was significantly lower than that of the control group. Similarly, there was a significant progressive increase in the TMA content in both the treatment and control groups as the storage time increased (Table 2).

### 3.3. Determination of Lipid Oxidation in Refrigerated Fish

Lipid oxidation was measured at three levels (Table 3) [2,49]: primary (the peroxide content), secondary (TBA-i), and tertiary (FR) oxidation. All the lipid oxidation indices markedly increased with increasing storage time in the control and treatment groups. Compared with that of the control group, the peroxide content of the treatment group was significantly greater after 2 and 6 days of storage. The TBA-i did not significantly differ between the control and treatment groups; however, on day 6, the treated group had a greater average TBA-i than did the control group. Finally, evaluation of tertiary lipid oxidation compounds revealed significantly lower formation in the treatment group than in the control group at the end of the 6-day storage period (Table 3).

### 3.4. Determination of Lipid Hydrolysis in Refrigerated Fish

**Figure 2:**
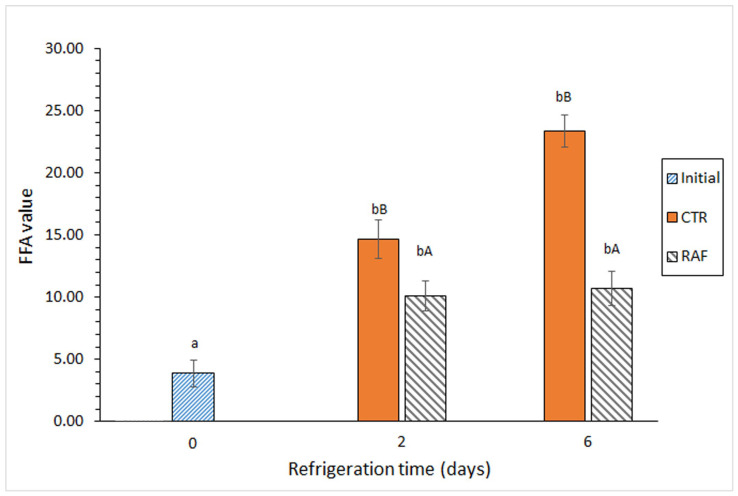
Evolution of the free fatty acid (FFA) value in refrigerated *Trachurus trachurus* subjected to different packaging conditions. Average values of three replicates (*n* = 3). Standard deviations are indicated by bars. For each packaging condition, different lowercase letters (a, b) indicate significant differences with respect to the refrigeration time; at each refrigeration time, different capital letters (A, B) indicate significant differences with respect to the packaging condition. The packaging conditions are shown in Figure 1.

There was a significant increase in the FFA content in both groups on day 2 of refrigerated storage (Figure 2). Notably, there was an additional increase in FFAs in the control group at the end of the 6-day storage period, whereas there was no significant increase in the FFA content in the treated group on day 6. A comparison between both groups revealed significantly lower FFA formation in the treated group than in the control group at both sampling times.

## 4. Discussion

### 4.1. Evolution of Microbial Development in Refrigerated Fish

On the basis of the microbial parameters (aerobic bacteria, psychrotrophic bacteria, and proteolytic bacteria counts) (Table 1 and Figure 1) and chemical indicators of microbial development (pH and TMA-N) (Table 2), the incorporation of *Gelidium* sp. flour in the gelatin packaging film significantly inhibited the development of some microbial groups in refrigerated *T. trachurus* muscle. However, no significant differences were found in *Enterobacteriaceae* bacteria. Although *Gelidium* spp. flour does not contain a single antimicrobial compound and the mechanisms of action of the potential antimicrobial compounds are different [50,51,52,53], its inhibitory activity typically does not affect equally all the microbial groups investigated. This result agrees with previous studies focused on the incorporation of other forms of *Gelidium* sp. in packaging films, such as an aqueous flour extract [38,39].

The addition of this aqueous extract inhibited the development of psychrotrophic bacteria, aerobic bacteria, and proteolytic bacteria and reduced the pH in refrigerated Atlantic mackerel (*Scomber scombrus*) [39]; this preservative effect could be observed during a 9-day storage period. Miranda et al. [38] evaluated aqueous flour extracts and reported their antimicrobial activity against *Enterobacteriaceae* (*Escherichia coli*, *Enterobacter aerogenes*, and *Klebsiella pneumoniae*), proteobacteria (*Vibrio alginolyticus*), *Bacillus cereus*, and *Bacillus subtilis*. Additionally, the presence of an aqueous *Gelidium* sp. flour extract in an icing system employed for the chilled storage of Atlantic mackerel inhibited the development of aerobic bacteria, psychrotrophic bacteria, *Enterobacteriaceae*, and proteolytic bacteria and led to lower chemical indices related to bacterial spoilage (pH, total volatile bases, and TMA) [38]. However, in the present study, we did not employ an aqueous extract; instead, the alga flour was added directly to the gelatin during the preparation of the packaging film. This constitutes a novel and potentially more practical application than those of previous works [38,39].

Several compounds present in red algae are known to be responsible for their antimicrobial effects. These include polysaccharides, polyunsaturated fatty acids, phlorotannins and other phenolic compounds, peptides and carotenoids [54]. Because a large variety of different polysaccharides exist in algae and are particularly complex, there is no single mechanism to which the antimicrobial effects of these polysaccharides can be attributed [54]. However, some authors have noted that the most likely antimicrobial mechanism is related to the presence of glycoprotein receptors on polysaccharides, which can bind to some compounds of the bacterial cell wall, cytoplasmic membrane, and DNA. Following this binding, the permeability of the bacterial cytoplasmic membrane increases, leakage of proteins from the bacterial interior occurs, and binding to bacterial DNA may even occur [55]. Polyunsaturated fatty acids can act by inhibiting the electron transport chain and enhancing oxidative phosphorylation activity in bacterial cell membranes. In this way, polyunsaturated fatty acids can affect adenosine triphosphate energy transfer and thus inhibit enzymes necessary for fatty acid synthesis within bacterial cells [55]. Peptides can inhibit or even kill bacterial pathogens via membrane permeabilization or interaction with phospholipids. In other cases, peptides can penetrate bacterial cells and introduce diverse harmful molecules into bacterial cells [50].

Phlorotannins have also been reported to produce antimicrobial effects by inhibiting oxidative phosphorylation and causing cell lysis by binding to enzymes and cell membranes [55]. Other phenolic compounds in red algae exert antimicrobial effects by disrupting the bacterial cell membrane, causing loss of cell integrity and thereby causing cell death [49]. With respect to carotenoids, their main antimicrobial mechanism leads to the accumulation of lysozyme, an immune enzyme that digests bacterial cell walls [54].

Previous research has shown that both polyphenols and polysaccharides from *Gelidium* have significant preservative effects [38]. Studies suggest that polyphenols contribute to antioxidant and antimicrobial properties, whereas polysaccharides enhance film-forming abilities and may also have antimicrobial effects [38]. Additionally, there is evidence supporting the synergistic interaction between polyphenols and polysaccharides, which could amplify their combined preservative effects [51,52]. This suggests that the interaction between these compounds can amplify their preservative properties, making them more effective when used together. Therefore, both polyphenols and polysaccharides are likely to contribute to the preservative effects of *Gelidium*, and their synergistic interaction should be considered an important factor.

In in vitro assays, El-Baroty et al. [56] reported the antimicrobial properties of *Laurencia papillosa* and *Galaxaura cylindrica* resulting from the presence of monosaccharides (mannuronic acid, galactose, and rhamnose). Glucuronic acid, arabinose, fructose, and glucose in water extracts of *Pterocladia capillacea* also showed antimicrobial activity in several in vitro assays [57]. Seedevi et al. [30] described a significant antibacterial effect of sulfated polysaccharides obtained from *Gracilaria corticata* against several human pathogens. Sulfated polysaccharides isolated from *Gelidium pacificum* were also reported to provide beneficial effects on mouse health by facilitating recovery of the gut microbiota [58]. In vitro analysis revealed an inhibitory effect of ethanolic and aqueous extracts of the red alga *Gelidium pusillum* on *Aeromonas caviae* growth [28]. The antibacterial activity of ethanol and aqueous extracts of red algae, including *Gelidium chilense,* against *Salmonella enteritidis*, *B. cereus*, and *E. coli* was also observed via different in vitro assays [37].

In seafood systems, the use of a *Gelidium corneum*–whey protein packaging film extended the shelf life of refrigerated (4 °C for 12 days) fish paste previously inoculated with *E. coli* O157:H7, *Listeria monocytogenes*, or *Salmonella typhimurium* [59]. Moreover, the growth of aerobic bacteria and psychrotrophic bacteria slowed in chilled Indian mackerel (*Rastrelliger kanagurta*) when a methanolic extract of the red alga *Gracilaria verrucosa* was included in the icing medium [31]. The use of aqueous and ethanolic *G. pusillum* extracts led to the 3-week survival of freshwater giant prawns (*Macrobrachium rosenbergii*), which was markedly longer than that of untreated prawns [28].

In terms of chemical indices related to microbial activity, endogenous enzymes and spoilage bacteria have been reported to decompose food proteins, leading to the formation of nitrogen-containing amines during fish storage [1,60,61]. In the present study, volatile amines such as TMA are likely produced by microbial enzymes (i.e., proteases) breaking down protein-derived molecules present in refrigerated fish muscle [1,57,58]; this effect increases in intensity as the storage time increases. This pathway explains the significant increase in the TMA content in the control and treatment groups (Table 2). Notably, in the present study, a preservative effect on protein degradation was inferred to be a consequence of the incorporation of *Gelidium* sp. flour in the packaging film.

Researchers have reported that algal extracts inhibit the increase in several chemical indices related to microbial development in seafood systems during refrigerated storage. The addition of a methanolic extract of the red alga *G. verrucosa* to the ice used to store Indian mackerel (*R. kanagurta*) inhibited the TMA, total volatile base, and biogenic amine contents due to the presence of several antimicrobial compounds in the algal extract [31]. There was an inhibitory effect on the TMA content and total volatile and biogenic amine formation, a result that was linked to the presence of substances such as butylated hydroxytoluene, sulfurous acid, heptadecane, mono(2-ethylhexyl) phthalate, and 1,2-propanediol. Moreover, the presence of an aqueous extract of *Gelidium* sp. flour in the icing system employed for the chilled storage of Atlantic mackerel (*S. scombrus*) led to the inhibition of TMA and total volatile base formation and to a lower pH [38].

### 4.2. Evolution of Lipid Oxidation in Refrigerated Fish

A preliminary sensory analysis was conducted to evaluate seaweed meal–gelatin ratios ranging from 1:99 to 15:85. The analysis was carried out by a trained panel of five judges from our laboratory, each with over 20 years of expertise in sensory evaluation. A seaweed meal–gelatin ratio of 5:95 represented the highest proportion of seaweed meal that neither produced a noticeable odor nor adversely impacted the physical appearance of the fish muscle portions.

The presence of the algal flour in the gelatin-based film significantly increased the peroxide content and significantly reduced the formation of fluorescent compounds. The conventional kinetics of lipid oxidation involve the progressive formation of primary and secondary lipid oxidation compounds and the subsequent interaction with nucleophilic compounds present in the fish muscle [62]. Thus, the detected peroxide value would be the balance of two opposite effects, i.e., formation because of the addition of oxygen to double bonds and a decrease in content because of breakdown into carbonyl compounds and interaction with nucleophilic compounds present in the fish muscle [63]. This damage mechanism is a multistep process that gives rise to a wide range of molecules [2,60]. Such molecules formed at the earliest stages are relatively unstable—susceptible to breakdown—and thus lead to the formation of lower-molecular-weight compounds (i.e., secondary oxidation compounds such as carbonyl compounds) [64,65]. Then, at advanced stages of oxidation, peroxides and carbonyl compounds (i.e., electrophilic molecules) react with nucleophile-type molecules with -NH_2_ and -SH groups present in fish muscle. As a result of this interaction, fluorescent compounds are produced, with notable losses in the sensory and nutritional value of the food [48,63]. Considering this basic development of the lipid oxidation mechanism, it can be concluded that the presence of the algal flour in the gelatin-based packaging system in the present study led to greater retention of primary lipid oxidation compounds than in the control samples. Hence, there was less formation of secondary oxidation products such as aldehydes and an overall preservative effect against lipid oxidation.

Researchers have already shown that aqueous *Gelidium* sp. flour extract has antioxidant properties. In a heated fish muscle system, the addition of an aqueous algal flour extract led to increased retention of primary and secondary lipid oxidation compounds (i.e., conjugated dienes and trienes, peroxides, and TBARS) [41]. Consistent with the present study, those authors also reported lower levels of compounds formed from the interaction between primary and secondary lipid oxidation compounds and protein-like molecules (i.e., fluorescent compounds). Moreover, there was greater retention of polyunsaturated fatty acids (PUFAs) in the samples enriched with the extract than in the control samples. López et al. [39] demonstrated that the incorporation of an aqueous extract in a gelatin film during refrigerated storage (4 °C for 9 days) of *S. scombrus* inhibited the formation of peroxides, TBARS, and fluorescent compounds in refrigerated fish and increased the retention of PUFAs.

In the present study, no subsequent analyses were carried out to explore the nature of the molecules responsible for the antioxidant effect of the algal flour. However, previous research has revealed several constituents that are responsible for antioxidant behavior depending on the extraction medium (e.g., water, ethanol, methanol, and ethyl acetate). In vitro studies considering red algae such as *Hypnea flagelliformis* [64], *G. verrucosa* [65], and *G. gracilis* [66] revealed the presence of phenolic compounds, flavonoids, carbohydrates, and alkaloids that are linked to such activity. Kim et al. [67] reported the antioxidant properties (based on 2,2′-azino-bis (3-ethylbenzothiazoline-6-sul-fonic acid) (ABTS) and 2,2-diphenyl-1-picrylhydrazy (DPPH) assays) of alginate-based films prepared by combining the red macroalga *Sargassum fulvellum* and black chokeberry. A wide range of in vitro studies have reported that carbohydrates from *Porphyra yezoensis* [29], *Gelidium corticata* [30], *Spyridia hypnoides*, *Asparagopsis taxiformis*, *Portieria hornemannii*, and *Centroceras clavulatum* [68] are responsible for antioxidant effects. Consistently, the inhibition of lipid oxidation in the present study could be explained by the notable presence of carbohydrate compounds in the algal flour (i.e., 42.8% total carbohydrates). Similarly, Ji et al. [69] analyzed and demonstrated the antioxidant properties of polysaccharides from the red alga *Porphyra haitanensis*. Moreover, bioactive trypsin-digested peptides obtained by in silico digestion of *P. haitanensis* have the potential to be employed as natural antioxidants for food stabilization [70].

Red algal extracts have also been employed to increase the quality of processed seafood. There was increased sensory acceptance of chilled Indian mackerel (*R. kanagurta*) after the inclusion of a methanolic extract of *G. verrucosa* in the icing medium; this effect was confirmed and linked to the presence of a high total polyphenolic compound content in the methanolic extract on the basis of the DPPH and ABTS assays [31]. Finally, there was less lipid oxidation and greater retention of endogenous antioxidants (i.e., astaxanthin and tocopherols) in cooked salmon paste by soaking in aqueous extracts of *G. chilensis*, *I. larga, G. chilense*, *G. radula*, *G. chamissoi*, and *G. skottsbergii* [37]. This preservative behavior was justified by the identification of polyphenols, carotenoids, phlorotannins, diterpenes, and phytosterols in the extracts.

### 4.3. Evolution of Lipid Hydrolysis in Refrigerated Fish

In the present study, the incorporation of the algal flour in the gelatin film inhibited FFA formation in refrigerated fish muscle (Figure 2). In contrast, Barbosa et al. [71] reported an increase in the FFA content in a heated fish muscle system as the addition of an aqueous extract of *Gelidium* spp. flour increased. The FFA content also increased in frozen *T. trachurus* when an aqueous extract of the present algal flour was included in the glazing system; as in the study by Barbosa et al. [71], FFA retention increased as the flour extract concentration increased.

During the refrigerated storage of fish, FFAs are formed due to the activity of microbial and endogenous enzymes [1,2,72]. During the initial storage period and before the end of the microbial lag phase, lipid hydrolysis is mostly the result of endogenous enzymes (i.e., lipases and phospholipases). The predominant mechanism of FFA formation subsequently involves microbial extracellular lipases. Considering the detection of strong lipid hydrolysis from day 2 to day 6 of refrigerated storage, it can be assumed that microbial activity is the most important pathway responsible for FFA formation. Therefore, the inhibition of FFA formation in the treated group can be explained by the abovementioned inhibitory effects on the growth of aerobic bacteria, psychrotrophic bacteria, and proteolytic bacteria and the lower average number of lipolytic bacteria. This inhibitory effect could be explained by the presence of hydrophilic and lipophilic bioactive compounds that hinder lipolytic microbial activity during refrigerated storage in the algal flour. These results suggest that there was less interaction between the microbial lipolytic enzymes and the higher-molecular-weight lipids (i.e., phospholipids and triacylglycerols) present in the fish muscle [73,74].

Only a few previous studies have investigated the effects of red algae on lipid hydrolysis. Among them, contradictory results related to the influence of such natural substrates on FFA formation in processed seafood have been reported. In agreement with our results, Barbosa et al. [71] reported an inhibitory effect on FFA formation in chilled hake (*M. merluccius*) muscle when an ethanolic and aqueous extract of the red alga *G. gracilis* was included in the icing medium employed for refrigerated storage. In contrast, Babakhani et al. [75] reported enhanced FFA formation in chilled minced Atlantic mackerel (*S. scombrus*) if previous treatment with an aqueous extract from the red alga *Polysiphonia fucoides* was carried out.

Owing to the significant environmental inconveniences associated with the use of plastic as a packaging material, in recent years, there has been active research into its replacement by natural polymers such as chitosan, alginate, gelatin or mucilage [76]. However, all these natural materials have inherent disadvantages compared with plastics. For this reason, these compounds are not intended to completely replace synthetic polymers but rather to reduce the toxic effects of synthetic polymers and address issues such as moisture and oxygen barriers, for which natural polymers can be a real alternative to traditional packaging materials, such as those used in the food industry [77]. Other attractive alternatives are animal-based preservatives, such as lactoferrin [78,79]; animal-derived enzymes, such as lysozime [78]; and microorganism-produced bacteriocins [80], which have also proven valuable for natural food preservation.

This study has several limitations, such as the lack of investigations into mechanical/barrier properties. This work represents preliminary research investigating whether the inclusion methodology of *Gelidium* meal in the film achieves antimicrobial and/or antioxidant effects in fish. Research on the physical properties of the film will be carried out on the basis of the most industrially applicable methodology to increase the shelf-life of the fish product. Another limitation is that the compounds responsible for the antimicrobial and/or antioxidant activities have not been identified. The identification of such compounds is complex, as these activities are possibly caused not by a single component but by the activity of several different components, as previously discussed. However, advancements in the identification of such compounds in an unequivocal manner would imply an important complement that needs to be investigated.

Finally, it is also necessary to ensure that the action of the seaweed meal does not worsen the sensory qualities of the fish. Although previous work carried out by the same research group revealed no organoleptic damage in fish preserved with algae, this effect should be evaluated in the near future.

## 5. Conclusions

This direct incorporation of *Gelidium* sp. flour in a gelatin-based film prevented the loss of the quality of refrigerated *T. trachurus*. Compared with the control samples packaged in gelatin alone, those packaged in gelatin containing algal flour presented significantly lower microbial development (aerobic bacteria, psychrotrophic bacteria, and proteolytic bacteria) and significantly lower chemical indices related to microbial development (pH and TMA). With respect to lipid oxidation, there was significantly greater retention of peroxides and significantly lower formation of secondary oxidation products in refrigerated fish muscle subjected to the combined gelatine–flour packaging system. Additionally, there was significantly lower lipid hydrolysis development in this system.

This study presents the results of simple, practical, and valuable employment of *Gelidium* sp. flour, which is considered an algal waste.

This approach meets the current global interest in the search for natural sources of preservative compounds and the beneficial use of marine byproducts in a combined strategy of sustainable food technology. Moreover, the proposed strategy does not require previous extraction of bioactive compounds by means of any kind of solvent and can be applied directly. Further research focused on the optimization of the experimental conditions (i.e., the algal flour–gelatin and algal flour–fish muscle ratios) on the basis of response surface methodology and on the analytical study of the molecules responsible for the preservative effects is advisable.

## Figures and Tables

**Table 1:** Evolution * of microbial quality parameters (log colony-forming units [CFU] g^−1^ muscle) in refrigerated *Trachurus trachurus* subjected to different packaging conditions **.

Microbial Parameter	Packaging Condition	Refrigeration Time (Days)		
		0	2	6
*Enterobacteriaceae*	CTR	1.00 ± 0.00 a	1.00 ± 0.00 aA	2.00 ± 0.23 bA
	RAF	1.00 ± 0.00 a	1.07 ± 0.12 aA	1.69 ± 0.82 aA
Psychrotrophic bacteria	CTR	3.91 ± 0.47 a	4.68 ± 0.33 aB	6.35 ± 0.39 bA
	RAF	3.91 ± 0.47 a	3.22 ± 0.16 aA	6.13 ± 0.27 bA
Proteolytics	CTR	2.57 ± 0.56 a	3.33 ± 0.39 aB	5.95 ± 0.41 bA
	RAF	2.57 ± 0.56 a	2.84 ± 0.06 aA	5.43 ± 0.16 bA
Lipolytics	CTR	2.00 ± 0.00 a	2.20 ± 0.35 aA	3.90 ± 0.56 bA
	RAF	2.00 ± 0.00 a	2.10 ± 0.17 aA	3.65 ± 0.67 bA

* The mean ± standard deviation of three replicates. For each packaging condition, different lowercase letters (a, b) indicate significant differences with respect to the refrigeration time; at each refrigeration time, different capital letters (A, B) indicate significant differences with respect to the packaging condition. ** Packaging conditions as expressed in Figure 1.

**Table 2:** Evolution * of pH and trimethylamine (TMA) content in refrigerated *Trachurus trachurus* subjected to different packaging conditions **.

Chemical Index	Packaging Condition	Refrigeration Time (Days)		
		0	2	6
pH	CTR	6.11 ± 0.04 a	6.34 ± 0.04 bA	6.53 ± 0.12 cB
	RAF	6.11 ± 0.04 a	6.26 ± 0.08 bA	6.29 ± 0.02 bA
TMA (mg TMA-N·kg^−1^ muscle)	CTR	0.4 ± 0.1 a	2.6 ± 0.8 bA	16.4 ± 0.5 cB
	RAF	0.4 ± 0.1 a	2.4 ± 0.9 bA	10.5 ± 1.5 cA

* The mean ± standard deviation of three replicates. For each packaging condition, different lowercase letters (a, b, c) indicate significant differences with the refrigeration time; at each refrigeration time, different capital letters (A, B) indicate significant differences with the packaging condition. ** Packaging conditions as expressed in Figure 1.

**Table 3:** Evolution * of lipid oxidation (peroxide value, PV; thiobarbituric acid index, TBA-I; fluorescence ratio, FR) in refrigerated *Trachurus trachurus* subjected to different packaging conditions **.

Lipid Oxidation Index ***	Packaging Condition	Refrigeration Time (Days)		
		0	2	6
PV (meq active oxygen·kg^−1^ lipids)	CTR	3.54 ± 1.74 a	6.70 ± 0.25 bA	12.12 ± 2.35 cA
	RAF	3.54 ± 1.74 a	10.03 ± 0.84 bB	16.7 ± 1.43 cB
TBA-i (mg malondialdehy-de·kg^−1^ muscle)	CTR	0.36 ± 0.03 a	1.32 ± 0.40 bA	3.26 ± 0.56 cA
	RAF	0.36 ± 0.03 a	1.31 ± 0.23 bA	5.15 ± 1.38 cA
FR	CTR	0.54 ± 0.11 a	1.51 ± 0.83 bA	1.63 ± 0.16 bB
	RAF	0.54 ± 0.11 a	1.54 ± 0.37 bA	1.23 ± 0.17 bA

* The mean ± standard deviation of three replicates. For each packaging condition, different lowercase letters (a, b, c) indicate significant differences with the refrigeration time; at each refrigeration time, different capital letters (A, B) indicate significant differences with the packaging condition. ** Packaging conditions as expressed in Figure 1. *** Abbreviations: PV (peroxide value), TBA-i (thiobarbituric acid index), and FR (fluorescence ratio).

## Data Availability

The original contributions presented in the study are included in the article; further inquiries can be directed to the corresponding author.
